# Retaining doctors and reducing burnout through a flexible work initiative in a rural South African training hospital

**DOI:** 10.4102/phcfm.v13i1.2799

**Published:** 2021-03-23

**Authors:** Rachel Schaefer, Louis S. Jenkins, Zilla North

**Affiliations:** 1 Department of Family and Emergency Medicine, Faculty of Medicine and Health Sciences, Stellenbosch University, Cape Town, South Africa; 2 Primary Health Care Directorate, Faculty of Health Sciences, University of Cape Town, Cape Town, South Africa; 3 Department of Medical Management, George Hospital, George, South Africa

**Keywords:** part-time posts, resilience, burnout prevent, training, flexible work, retention

## Abstract

**Background:**

South African doctors work up to 60 h per week to ensure 24-h service delivery. Many doctors are physically and emotionally exhausted, neglecting families, self-care, patient empathy and innovative thinking about complex health issues. Exposure to clinical work hours demonstrated a dose effect with burnout, suggesting cause and effect, affecting up to 80% of doctors. To retain good doctors, their complex needs must be recognised and allowances made for flexible work options.

**Taking a risk:**

George Hospital, a large regional training hospital in a rural district, converted some full-time medical officer posts to part-time posts. This was in response to doctors’ requests for more flexible work options, often after returning from maternity leave or in response to burnout. Perceived risks revolved around institutional resource security and that part-time post vacancies would be difficult to fill.

**Reaping the benefits:**

Employing doctors in part-time posts has created stability and continuity in the health team. The hospital had generated a cohort of young professionals who care with empathy and have emotional resilience to train others and plough their skills back into the healthcare service.

**Conclusion:**

Reducing working hours and creating flexible options were concrete ways of promoting resilience and retaining competent doctors. We recommend that training and work of doctors be structured towards more favourable options to encourage retention, which may lead to better patient care.

## Background

Globally, there is a shortage of doctors, which is accentuated in developing countries.^1^ According to the World Health Organisation (WHO), South Africa (SA) had 9.1 medical doctors per 10 000 population in 2017, in comparison with the average global density of 15 per 10 000 people.^1,2^ This is in contrast with other BRICS countries, for example Brazil with 21.5, China with 17.8, Russia with 40.1 and India with 7.8 doctors per 10 000 population.^1^ In SA, this shortage is aggravated by the large private or public healthcare divide, with 30% of doctors caring for 85% of the population in the public sector.^3^ The rural or urban divide contributes further to this shortage, where 46% of the country’s population living in rural areas are being cared for by 12% of doctors and 19% of nurses.^4^ In the Garden Route district 3.3 doctors per 10 000 population care for the roughly 550 000 people in the public sector (Western Cape Department of Health Human Resources Department staff approved post list as at April 2020).

Because of this shortage and the need to cover 24-h services, full-time doctors are contracted to work overtime, amounting to a 56-h work week. This effectively translates into working 7 days a week, 8 h a day. Consequently, many doctors are physically and emotionally exhausted. Family life, self-care, patient empathy and thinking innovatively about complex health issues have become remote realities divorced from daily clinical work. Subsequently, many doctors face burnout and aim to leave full-time medical practice to find sustainable solutions to their dilemma, wanting to be a good doctor on the one hand, whilst also wanting a healthy personal and family life on the other hand. A strong theme identified as a reason why doctors stay to work in rural areas in SA was having a healthy work-life balance.^5^ This is in keeping with Generation X and Y worldwide, where a work-family life balance is more important than hard work.^6^ Motivational drivers of Millennials, who are today’s doctors, include flexibility, work-life balance, convenient social relationships, need for coaching-based leadership and the opportunity to develop.^7^

Whilst African countries are struggling to retain sufficient health professionals, some developed countries have identified initiatives to improve work-life balance, including flexible work hours, telework (working from home or a satellite location), job sharing (sharing a full-time job between two employees), voluntary reduced work hours, parental leave and financial and/or informational assistance with childcare and eldercare services.^8^ Further research has been called for to evaluate how improved work-life balance practices can influence organisational performance, including enhanced social exchanges, improved performance and reduced turnover. However, the impact of these processes may be moderated by a number of factors, including national context, post level and management support.^9^ The national Department of Health in the United Kingdom launched the ‘Improving Working Lives’ (IWL) scheme in 2000. The Improving Working Lives Standard emphasises that every staff member in the NHS is entitled to work in an organisation that demonstrates its commitment to give staff greater flexibility and control over their own time, for example improved access to childcare and carers support and the compressed hours scheme, where staffs are enabled to extend their working day into the evening, accommodating evening clinics and home visits for working patients who are home at night.^10^

The focus of this opinion paper is to describe one such initiative in a South African training hospital where the authors implemented flexible working hours, with the aim to prevent burnout and retain good doctors. We hope to stimulate a strong debate in Sub-Saharan Africa towards this aim.

## Burnout revisited

**FIGURE 1:**
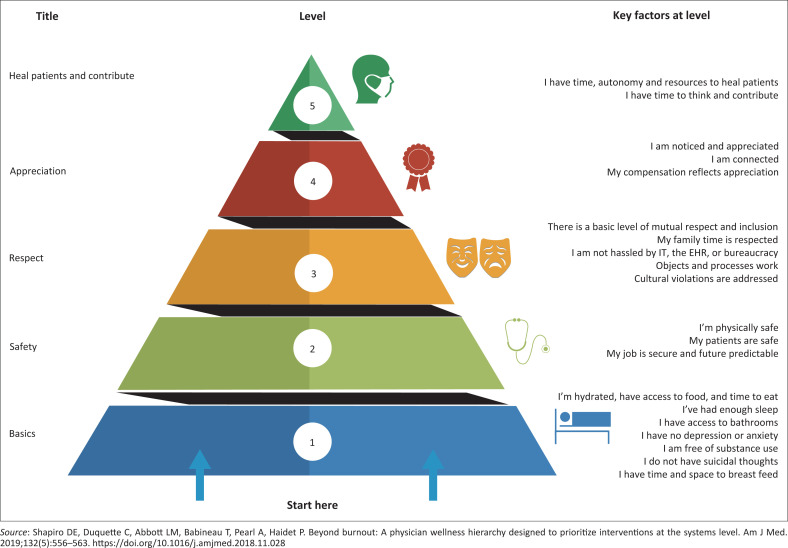
An adaptation of Maslow’s hierarchy of needs to physician’s needs.

Physician burnout has been the topic of multiple international publications and conversations.^11^ Burnout, a syndrome of emotional exhaustion and depersonalisation, resulting from the workplace, has been reported to affect 54.4% of doctors in the USA between 2011 and 2014.^12^ Exposure to clinical work hours demonstrates a dose effect with burnout, suggesting cause and effect.^12^ The situation is similar in SA, with studies in the urban and rural areas in Cape Town,^13,14^ the Orange Free State^15^ and at Wits University^16^ indicating 80% of registrars suffered from burnout. An adaptation of Maslow’s hierarchy of needs to physicians’ needs indicates that healing of patients only becomes important after other basic needs are fulfilled (See Figure 1).^17^

This model of hierarchy suggests that addressing burnout needs to happen at multiple levels: workshops, training, time off, etcetera, as singular interventions are insufficient. Burnout was the factor most strongly related to doctors’ plans to reduce their working hours to part-time within 1 year, leaving clinical practice completely, or re-routing to a different practice.^18^ In rural South Africa, this translates to leaving public practice for private, leaving rural areas for urban or leaving South Africa for other countries. Doctors in large hospitals are typically employed in departments, work fixed hours with fixed overtime, in a rigid routine involving ward work, outpatient clinics, theatre, outreach and teaching. They usually do not have the options, authority or the skills to resolve their dilemma of being ‘stuck’ in a pattern that makes them vulnerable to burnout.^19,20^ Organisational leadership is needed to change the status quo. The current, worldwide Covid-19 crises have rekindled the heated debate of healthcare workers around burnout.^21^ We need a re-alignment between the needs of caregivers and those of the healthcare system. Key concepts relating to intrinsic work motivation include autonomy, competence and relatedness.^21^ To retain good doctors, we must recognise their competence, allow flexible working hours and ensure on-going relatedness to the health team.

## Setting

George Hospital is a regional hospital in the Garden Route district of SA. It has 272 beds, eight general specialities, a 6-bed high care unit, an emergency centre (EC) and six theatres. It serves an outlying population of 620 000 people and reaches out to 10 district hospitals and various primary healthcare (PHC) clinics.^22^ The Department of Family and Emergency Medicine governs the EC, which manages 140 patients daily, as well as outpatient clinics, theatres and a ward. The department delivers the ‘district hospital’ service, caring for patients with undifferentiated illnesses and coordinates inter-professional collaboration. There are six full-time medical officers, three community service medical officers (COSMOs), six part-time doctors and four consultants. The Garden Route training complex hosts postgraduate registrars in Family Medicine from the University of Stellenbosch at three rural district hospitals. Registrars in other disciplines from the University of Cape Town (UCT) are trained at George Hospital. Recently, UCT introduced a longitudinal integrated clerkship for final year medical students. Whilst this initiative was spearheaded within the Department of Family Medicine, the principles and processes are applicable in many similar contexts.

## Taking a risk

During the previous 5 years, some full-time medical officer (MO) posts were converted to part-time temporary contract posts. This was in response to doctors’ requests to work more flexible working hours, or to stop overtime entirely. These requests often came after returning from maternity leave or in response to burnout. The perceived risks related to creating contract posts revolved around resource security for the clinical department. The hospital has an approved post list for each department. Part-time budgets (sessions) and overtime budgets are separate funds that are allocated yearly and are not department-specific. These contracts are temporary, fluid and renewed yearly. (‘Sessions’ relate to the number of hours that are contracted per week. For example, a 12-h sessional contract would entail a 12-month contract to work 12 h per week). Whilst this allows for the ability to respond to needs, it is also a budget at risk of being cut, or forfeited to priority needs in other clinical departments, or underspent and hence removed from the budget completely. This is not dissimilar to most large public hospitals in SA. Furthermore, locum expenditure (which is not clinical department-specific) was initially translated to sessional posts to first test the processes before considering removing overtime from posts and transferring funds across budget lines. Whilst this seemed like a simple thing to do, it was not institutional culture and involved a large administrative process. Another perceived risk was that whilst doctors would commit initially, as their personal circumstances change, some could resign from their posts, with the difficulty of filling a 16-h per week sessional post (See Table 1).

**TABLE 1:** Perceived risks and benefits of part-time posts for the health service.

Perceived risks		Perceived benefits
1	Part-time staff unwilling to work unsociable hours: Not able to cover after hours’ services (where the doctor committed to a few hours per week is not committed to working weekends or nights).	Increased flexibility of staff who works less hours, allowing for sacrifices to work some unfavourable shifts.
2	Difficult recruitment of unattractive posts.	Part-time staffs are adaptable and flexible to cover overtime slots during staff shortages and at short notice.
3	Administrative complexity regarding rostering, leave and training.	Improved staff retention with lower turnover.
4	Loss of posts in the department	Improved emotional resilience resulting in better patient care and teamwork
5	Loss of unallocated overtime budget	Stability and continuity in the health team and consequently in the health service.
6	Under spending on staffing can result in a budget cut on staffing.	Senior doctors can cross cover in other departments due to broad skills and knowledge base.

The part-time posts were welcomed by doctors and unexpectedly easier to fill than was initially anticipated. More MOs, particularly females, and increasingly male doctors who were committed to continue working in the district health system, were requesting flexitime options. Two further MO posts were converted to part-time posts and five doctors committed to working part-time for 12–20 h per week each. Subsequently, these annual contracts have been secured as 3-year contract posts, creating stability for the individuals and the department.

## Reaping the benefits

**FIGURE 2:**
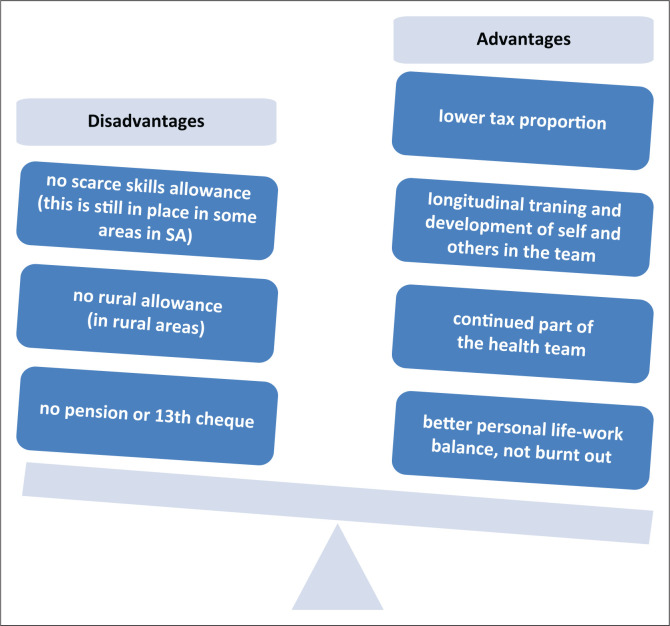
Balancing disadvantages and advantages of part-time posts for the individual.

Looking back over 5 years, the authors are convinced that there have been more benefits than risks to the doctors and the health service. Many perceived risks are outweighed by the benefits, and the advantages to the individual often translate into benefits for the health system and consequently to patients (See Figure 2).

Because they work less hours, these doctors often come to work more energised, focused and willing to take on other roles like student, interns and nurses’ teaching, clinical and corporate governance and managerial roles. Employing MOs in part-time posts has created stability and continuity in the health team. We have generated a cohort of young professionals who are not burnt out, are eager to serve, give care with empathy and have emotional resilience to also invest in the training of others. The advantage of having fully engaged clinicians was initially underestimated by the consultants. Most of these doctors previously occupied full-time posts and subsequently developed insight in the difference in quality of service delivery which they were providing. Put differently, doctors did not realise how burnt-out they were till the moment of retrospection, and this has helped in their developing a better sense of loyalty to the hospital. The clinical department has not experienced the high turnover of posts that was expected. The hospital has retained many of these doctors for many years now. The flexitime posts have also become sought after by other clinical departments, hoping to find a more balanced work-life in rural public practice within a state hospital or PHC clinic. The box below gives the testimonies from two doctors that illustrate these points.

The lessons learnt to retain and integrate part-time staff include making them feel part of the health team by:

1. giving them clinical, governance and teaching responsibilities
2. including them in training events and team days and
3. ensuring that they participate in the staff performance evaluation processes.

This is not unlike recommendations for part-time doctors in the United Kingdom.^23^ We slowly changed the traditional view of these doctors, who are often viewed as temporary staff, working in isolation, doing mostly clinical work and functioning outside the team. In reality, the turn-over of staff on the temporary contracts has been less than the full-time permanent contracts.

In creating a favourable, working environment by decreasing the expected hours, relationship building, collaborative leadership and active management of the posts (active and timely recruitment), these doctors have been retained in service. Consequently, they have entered a more favourable mental space, feel valued in the team and are able to organise their lives better. They are working in the frontline in family and emergency medicine, often unusual hours, with patients who expect much and often need much care.^24^ They perform at a high level of clinical care, get time to exercise and rest, do not suffer from physical and emotional exhaustion and the female doctors with babies get time to breastfeed.

BOX 1 Testimonies from two doctors.

‘I have been working in the government sector since 2012 (8 years). My sessional contract started May 2016 until the present. The reason why I choose to do sessional work instead of a full-time MO position is mainly to attempt a healthy balance between work life and family life. I feel the need to be stimulated in my occupation, but at the same time I don’t want to look back at my life wishing I spent more time with my child and husband. Working in an emergency unit is very stimulating and one is always learning something new, but it can also become physically and emotionally draining if it is done full-time. Doing sessional work in an emergency unit also helps to prevent EC burnout, therefore making it more sustainable to do EC work long-term. I suppose the downside of sessional work is that it is often not the best career move, especially if one wants to specialise in the future.’

(Sessional medical officer in the Family Medicine department, mother and sportsperson)

‘After the birth of our firstborn, when I started working again, I took a sessional post in a primary healthcare clinic as I wanted to be a more active presence in her life. I was not the main financial provider for our family, allowing me a level of freedom. This continued for three years until the illness of my second child forced me to resign completely. While working in the clinic, even though I was only a sessional doctor, I could make it my own. I really felt like the community’s doctor. I got to know the patients and staff well and enjoyed the stability of the clinic immensely. Working sessions, I could plan my week and initiate process improvements during my off times. I used this time to establish a booking system, an NGO focusing on food gardens for the area, and a couple of collaborative community activities. I could also work in the clinic’s food garden because every hour was not crammed into clinical work. At home I could be present in my children’s lives, intervene when issues arose and stay in touch with their developmental needs. I would have not been able to do this if I was permanently absent during their awake hours. However, the work in the clinic was very isolated. I did not get to know a peer group that I could relate to or consult. I did not learn to understand the bigger system and how to solve systemic problems, which frustrated me. As sessional doctor, I was excluded from training, staff performance management and decision-making. I was surprised by the overarching experience from my supervisor that by choosing to work sessions, my exclusion was inevitable and acceptable. Years later, as the medical manager at George Hospital, I worked actively with the family medicine department to proactively avoid these negative experiences that sessional staff could have’

(Previous sessional medical officer in PHC, now medical manager)

## Conclusion

Creating more flexible working options are concrete ways of reducing burnout, encouraging resilience and retaining competent doctors, which in turn, maintains continuity of care and training within the health team. We support the previous calls of health leaders to structure the training and work environment of doctors towards more favourable options to encourage retention and reduce burnout, ultimately leading to better and safer patient care. The hope is that more of these innovations will be implemented in similar contexts in Africa.
